# Validation of the SPF-Q, an instrument to assess the quality of production functions to achieve well-being, among multimorbid patients

**DOI:** 10.1186/s12955-020-01573-z

**Published:** 2020-10-01

**Authors:** Anna Petra Nieboer, Jane Murray Cramm

**Affiliations:** Department of Socio-Medical Sciences, Erasmus School of Health Policy & Management, Erasmus University Rotterdam, Rotterdam, The Netherlands

**Keywords:** Quality of production functions, Social production functions, Production needs, Substantive needs, Multimorbidity, Control, Loss, Efficiency, SPF-Q

## Abstract

**Background:**

In a time of ageing populations, examination of the ability of multimorbid patients to achieve well-being has become increasingly important. Social production function theory is used to characterise people’s ability to achieve well-being. Whereas much research has examined the fulfilment of substantive needs, far less research has investigated the quality of production functions (*being in control*, *avoiding a loss frame*, and *efficiency*) to achieve well-being. Therefore, this study involved the development and validation of the Social Production Function-Quality of production functions (SPF-Q) instrument to assess the quality of production functions via the fulfilment of production needs to achieve well-being.

**Methods:**

The 12-item SPF-Q was used to assess the quality of production functions via the fulfilment of production needs to achieve well-being among patients with multimorbidity from seven health care practices in the region of Tilburg, the Netherlands. A total of 216 patients filled in the questionnaire (55% response rate). To test the validity of the SPF-Q, we used structural equation modelling to specify a measurement model by loading each item on its respective latent factor, and we examined associations between production needs and other measures.

**Results:**

Psychometric results clearly showed that the SPF-Q is a valid and reliable instrument for the assessment of the quality of production functions among multimorbid patients. Confirmatory factor analyses revealed good indices of fit for the instrument. As indicated by the high reliability coefficient, the scale also showed good internal consistency. We found support for construct validity through significant positive correlations between substantive and production well-being needs, as well as with overall well-being and life satisfaction. Moreover, production needs added to multimorbid patients’ overall level of well-being in addition to the substantive needs.

**Conclusion:**

This study clearly showed that the SPF-Q is a valid and reliable instrument for the assessment of production needs among multimorbid patients. Given that multimorbidity is becoming the leading threat to population health, such an instrument can help to improve the ability to achieve well-being in this vulnerable population.

## Background

Ageing populations, unhealthy lifestyles (smoking, physical inactivity, unhealthy diet), and new and improved diagnostic techniques and treatments that enable people to survive once-fatal diseases are major contributors to the enormous increase in the number of people with more than one chronic non-communicable disease [1]. More than 50 million European citizens currently have multimorbidity (i.e. at least two chronic non-communicable diseases), and the Organisation for Economic Co-operation and Development predicts that this number will increase dramatically in the near future [2]. Multimorbidity increases mortality rates and strongly impairs the quality of life and well-being of affected individuals and their families [3]. The well-being of multimorbid patients is thus becoming an urgent topic of policy and economic debates, and its improvement is emerging as a key societal aspiration, as it is expected to relieve burdens on health care systems [4].

While coping with the consequences of their chronic diseases and changes in circumstances, multimorbid patients make diverse efforts to improve their situations in various aspects of life, with the ultimate aim of achieving overall well-being [5]. Research shows that chronic illnesses, functional limitations, and disabilities do not affect patients’ well-being in the same manner [3, 6]. People’s resources (e.g. certain assets, income, having a spouse and children) influence well-being in times of poor as well as good health [7, 8]. Social production function (SPF) theory, developed by Lindenberg [9, 10], provides a full characterisation of people’s ability to achieve well-being. The approach taken by SPF theory is that need satisfaction can best be viewed in terms of production functions. A particular level of need satisfaction (the output) is “produced” by a particular input. According to SPF theory, people are producers of their own well-being in terms of need fulfilment [10]. Drawing on economic and psychological theories, SPF theory sees humans as active agents who self-regulate to choose cost-effective ways to produce well-being. People influence their own well-being, by and large in a way that is positive for their well-being, albeit not always for their well-being in the long run [10]. For example, some unhealthy behaviours provide comfort in the short run (such as eating unhealthy comfort foods or being inactive), but are detrimental to a person’s physical well-being in the long run.

People’s need to produce well-being is covered by substantive needs (e.g. *affection*) and production needs (e.g. *control*) [10–12]. From a self-regulatory point of view, separate consideration of these needs is useful [13], given that the ability to determine how certain circumstances affect multimorbid patients’ ability to achieve overall well-being, and which of the underlying substantive and/or production needs are not fulfilled, may help to determine the changes required to protect their well-being.

### Achieving well-being via fulfilment of substantive needs

According to SPF theory, people coping with multiple chronic diseases still make diverse efforts to improve their living conditions with the overall aim of achieving physical and social well-being [9, 14, 15]. Lindenberg [9] identified five substantive needs which must be fulfilled to some extent to achieve well-being: *comfort*, *stimulation*, *status*, *behavioural confirmation*, and *affection*. Physical well-being can be achieved by (1) being in a situation represented by optimal *comfort* and (2) creating the right amounts of mental and physical *stimulation*. Social well-being is achieved by (1) having a certain amount of *status* based on one’s occupation, lifestyle, or talents; (2) living according to certain values and norms (*behavioural confirmation*); and (3) receiving enough *affection* through friendship, intimacy, and emotional support [10, 16, 17]. Physical and social well-being are achieved on the way to the ultimate goal of overall subjective well-being (optimal quality of life or mental well-being) [16, 17]. In the hierarchy of SPF theory, substantive needs fall below the two ultimate needs (social and physical well-being) and above lower-order (resource-related) needs required for their production (activities and resources such as income, health care system, social network) [10, 17–19]. Production functions specify the factors needed to fulfil a certain need. Thus, an array of production functions between needs on different levels shows how well-being is generated, maintained, and changed. By recognising this hierarchy, we can better understand the impacts of chronic illnesses and accompanying functional limitations, and thereby determine the types of care and support that multimorbid patients require [14]. Many researchers (e.g., [13–15, 17–20]) have studied and described the five substantive needs in depth.

### Achieving well-being via fulfilment of production needs

Although SPF theory specifies needs in the sense of what people want to realise (or obtain), it does not specify goals enabling such realisation and fulfilment of these needs. It is, however, not only important to understand *what* people produce—social and physical well-being—but also *how* they produce it (i.e. the quality of their production functions) [20]. Thus, whereas the goals serving the fulfilment of substantive well-being needs (to which the discussion in the literature thus far has been restricted) have to do with production levels or output, production well-being needs involve the production process [20]. Lindenberg (e.g. [9, 21]) has repeatedly emphasised the importance of production need fulfilment (i.e. people’s needs and preferences as to how they structure their lives) for the realisation of well-being. Production needs, for example, involve the degree of autonomy or control that people have, the availability of multi-functional activities and resources leading to the efficient production of well-being, limited vulnerability or the evasion of loss, and consistency or avoidance of moral conflict [9, 10, 20]. Among people with similar resources (e.g. spouse, university degree, high income level, access to the same health care facilities, coping with the same diseases), those who feel in control, are not caught in a loss frame, and are efficient with their time have higher-quality production functions and thus are better able to achieve well-being. *Control*, *avoidance of a loss frame*, and *efficiency* are considered to be especially important for multimorbid patients with fewer resources to fulfil substantive needs.

#### Being in control

Van Bruggen [20] clearly showed the importance of the extent to which people perceive that they are in control of their own actions and processes related to the realisation of well-being. Such control facilitates the production of well-being, whereas perceived loss of control over one’s own life (e.g. as a result of coping with multiple chronic diseases) impedes the (re)construction of the SPFs that a person desires. The belief that one can exert control over stressful events, such as coping with the consequences of multiple chronic illnesses, has long been known to help people cope with stress [22], as people who have a sense of personal control seem to be better off than those who do not feel in control [23]. Coping with the consequences of multiple chronic diseases is complex and has multiple implications in various spheres of everyday life; multimorbid patients face many *physical* and *social* losses [24, 25]. The physical limitations that their health imposes (e.g. respiratory problems, joint pain, lack of muscular endurance) affect their ability to perform many daily activities (*comfort*, *stimulation*) and to invest in social relationships (*affection*). Multimorbid patients who retain control can more successfully fulfil their substantive physical and social well-being needs than can those who feel less in control.

#### Avoiding a loss frame

Nieboer [7] clearly showed that the loss frame, in addition to substantive well-being goals, influences people’s production capacity and is a crucial predictor of overall well-being. Being caught in a loss frame increases a person’s vulnerability because it incapacitates his or her adjustment to changing circumstances and protection of resources. Loss can seriously affect the ability to react to deterioration in the quality of one’s production functions. Research shows that the well-being of multimorbid patients depends on their ability to maintain an optimistic view of life and positive state of mind, rather than becoming caught in a loss frame [26, 27]. Multimorbid patients who hold more negative thoughts about the future and focus more on the things that they keep losing in life report poorer well-being outcomes than do those holding more positive views. Almost all multimorbid patients deal with constant pain and physical deterioration, but those who are optimistic and have a positive state of mind force themselves to see life from a different perspective [22, 26]. They consider that they should not complain about relatively unimportant matters, but rather invest in social relationships and in holding more positive thoughts and an affirmative attitude toward life; they explained that one way to achieve joy in life was to be surrounded by people with whom one could share positive thoughts or memories. In times of trouble or worry, they immediately took action by simply phoning someone. Patients caught in loss frames were less able to effectively adjust to changing circumstances, did not protect their resources, and reported more negative thoughts about the future, such as worry about their financial situations, disease, and pain, which reduced the quality of their production functions. Instead of trying to find ways in which to deal with the consequences of chronic diseases through balance with positive factors (e.g. *affection* through social contacts; *status*, *behavioural confirmation*, and *stimulation* through continued investment in things they are good at), they tend to become more isolated and stop investing in the fulfilment of their substantive well-being needs [26].

#### Being efficient

Under Lindenberg’s [9] assumption that people strive to maximise well-being, it follows logically that people want to produce well-being as efficiently as possible, maximising it by achieving the lowest possible cost–benefit ratio in production. Lindenberg calls attention to the use of multi-functional activities (e.g. sports and love making, which can simultaneously yield social and physical well-being) as one important means to achieve such efficiency. People tend to choose activities that yield multiple forms of well-being over those yielding only one form, reducing the cost–benefit ratio [20]. Multimorbid patients often have fewer possibilities for the production of well-being, and the ability to realise substantive goals declines with age. Thus, the efficient use of activities and investment to produce well-being becomes increasingly important. Patients who are better able to achieve *efficiency* in the production of well-being are expected to report higher levels of overall well-being.

### Study aims

Whereas much research has examined the fulfilment of substantive needs to achieve well-being, far less research has investigated the quality of production functions. Lindenberg and other SPF researchers discuss the importance of ‘production goals’ (e.g. [7, 9, 20, 21].), and Van Bruggen [20] further conceptualized the production needs in her work on the quality of production functions, but this was based on qualitative research only. If SPF theory is to serve as a framework for the examination of subjective well-being in quality of life studies, a means by which to measure the quality of production functions for the realisation of well-being is needed. Therefore, this study involved the development and validation of the Social Production Function-Quality of production functions (SPF-Q) instrument to assess the quality of production functions via the fulfilment of production needs (*being in control*, *avoiding a loss frame*, and *efficiency*) to achieve well-being.

To test the validity of the SPF-Q, we used structural equation modelling to specify a measurement model by loading each item on its respective latent factor, and examined associations between production needs and other measures. We expected that substantive and production needs would be related. More importantly, we investigated whether production needs added to multimorbid patients’ overall level of well-being in addition to the substantive needs, which would support the construct validity of the instrument. We validated the SPF-Q among multimorbid patients, given the great challenges that these patients face in the achievement of well-being.

## Methods

### Participants and procedure

We included patients with multimorbidity from seven health care practices in the region of Tilburg, the Netherlands. All patients enrolled in at least two chronic care programmes (combined diagnosis and treatment of diabetes, cardiovascular diseases/conditions, asthma and/or chronic obstructive pulmonary disease [COPD], age-related frailty) were selected from the practices’ data registries as eligible participants. No additional inclusion criterion was applied. Patients first received questionnaires at home via post. A few weeks later, reminder notices were sent to non-respondents. Another few weeks later, second reminder notices with duplicates of the questionnaire were sent. Finally, when telephone numbers were available, we called non-respondents to ask them to fill in the questionnaire. In total, 216 multimorbid patients filled in the questionnaire. Nineteen respondents were not eligible to participate due to incorrect addresses (n = 5); recent moves (n = 2); death (n = 4); terminal illness with admission to a hospice/nursing home (n = 2); and inability to fill in the questionnaire due to poor cognitive function (n = 2), recent stroke (n = 1), and poor eyesight (n = 3). Thus, the final response rate was 55%.

The medical ethics committee of Erasmus Medical Centre, Rotterdam, the Netherlands, determined that this research (study protocol no. MEC-2018–021) was not subject to the requirements of the Medical Research Involving Human Subjects Act. Written informed consent to participate in the study was obtained from all participants.

### Measures

#### SPF-Q

In the prior qualitative research of van Bruggen [20] the quality of production functions to achieve well-being have been richly described and conceptualized. We used this extensive qualitative study as preparation of the operationalization of production needs. It formed the foundation for the development of a new measurement instrument to assess the quality of production functions to achieve well-being. From this initial qualitative work, we generated a detailed listing of categories and themes [28]. Using a rigorous process and multiple types of triangulation [(1) placing the generating categories and themes in the literature regarding production needs being in control, avoidance of a loss frame and efficiency, (2) placing them into our own theoretical and empirical expertise on the subject, and (3) placing them into our own theoretical and empirical expertise on this patient population], important concepts and items were brought forward by each author and then discussed by both authors. This was an iterative process which led to the final 12-item SPF-Q to assess production needs in three (sub)dimensions: *being in control* (e.g. ‘Do you feel in control of your life?’, ‘Are you able to manage your life how you like?’), *avoiding a loss frame* (e.g. ‘Are you pessimistic about your future?’, ‘Do you see opportunities to turn your life in a positive direction?’), and *efficiency* (e.g. ‘Do you do things that are both fun and challenging at the same time?’, ‘Do your activities provide you with multiple benefits, such as fun, relaxation, and a social life?’). Response categories were never, sometimes, often, and always.

#### SPF_ILs

We used the 15-item version of the Social Production Function Instrument for the Level of well-being short form (SPF-ILs) [17] to assess whether respondents’ needs for *affection*, *status*, *behavioural confirmation*, *comfort*, and *stimulation* were fulfilled. Responses are provided on a 4-point scale, with higher scores indicating better experienced well-being. The reliability of the SPF-IL for the assessment of well-being has been verified in elderly populations [29, 30]. The Cronbach’s alpha value for the SPF-IL in this study was 0.86, indicating good internal consistency. Mean overall well-being and subscale scores were calculated for further analyses.

#### Overall well-being: life satisfaction

We measured overall subjective well-being with respect to life satisfaction using Cantril’s [31] ladder. This item (‘On a scale of 1 to 10, how satisfied are you with your life as a whole now?’) has been used widely and provides a general cognitive evaluation of a person’s overall well-being.

#### Socio-demographic variables

We collected data on participants’ age, gender, monthly net income, educational level, and marital status to enable description of the study population. Monthly net income, educational level, and marital status were dichotomised. Male gender, monthly net income ≥ €1350, elementary education or higher, and married/living together functioned as reference categories.

### Analysis

We used the following procedure to validate the SPF-Q. First, we used descriptive statistics to characterise the study population’s age, gender, marital status, educational level, income level, overall level of well-being, and life satisfaction. Second, we calculated mean scores, standard deviations (SDs), numbers of missing responses, and lambda values for the 12 SPF-Q items. Third, we used LISREL program version 8.80 [32] to conduct confirmatory factor analyses and verify the factor structure of the SPF-Q. We treated the data as ordinal and used robust DWLS estimation with polychoric correlations to fit factor models. The robust DWLS method has been recommended for ordinal data with 5 or less categories [33]. To test the measurement models, we used multiple imputation techniques using Expected Maximization algorithm. Fourth, we assessed model fit using the cut-off criteria of Hu and Bentler [34]: standardised root mean square residual (SRMR) < 0.08, root mean square error of approximation (RMSEA) < 0.06, and comparative fit index (CFI) > 0.95. A small and non-significant chi-squared statistic (Satorra–Bentler scaled chi-squared value) was seen as an indicator of exact model fit. However, due to its sensitivity to large samples, this statistic should be interpreted with caution. Fifth, we calculated Cronbach’s alpha values to assess the internal consistency of the subscales and investigated inter-correlations to verify conceptual relatedness among (sub)scales both for observed data and latent scores. Moderately strong associations between subscales point to related but distinct concepts (discriminant validity). We also computed a composite reliability index (CRI) based on the factor loadings of the first-order constructs to assess overall scale reliability. Finally, we assessed the construct validity of the SPF-Q (for the underlying dimensions of *control*, *avoidance of a loss frame*, and *efficiency*) by analysing associations of the latent scores of the production needs with *affection*, *behavioural confirmation*, *status*, *comfort*, *stimulation*, overall well-being, and life satisfaction. We also conducted multivariate regression analyses to test whether these production needs explained life satisfaction in multimorbid patients in addition to the contributions of substantive needs.

## Results

### Descriptive results

Table 1 shows descriptive characteristics of the sample of multimorbid patients. Patients’ mean age was 77.42 ± 10.63 (range 50–98) years; 59.1% were female, 43.2% were single, 40% had low educational levels, and 37.1% had low incomes. Multimorbid patients are known to have lower levels of well-being and life satisfaction [35–37]; in accordance, we found low levels of overall well-being (2.65) and life satisfaction (6.90) in this sample compared with scores of 2.82 (SD 0.35) for well-being and 7.66 (SD 1.098) for life satisfaction in a general adult population [17].

**Table 1:** Characteristics of multimorbid patients (n = 216)

	Mean (standard deviation) range or percentage
Gender (female)	59.1%
Age (years)	77.42 (10.63)
Marital status (single/widowed)	43.2%
Education (low)	40.0%
Income (low)	37.1%
Well-being	2.65 (0.51) 1–4
Life satisfaction	6.90 (1.36) 1–10

### SPF-Q item characteristics

Table 2 displays characteristics of the 12 SPF-Q items. Item non-response (missing) rates ranged from 1 to 2%. Approximately 92% of participants responded to all items. All items had loadings > 0.60 on the intended factors.

**Table 2:** Characteristics of the SPF-Q items (n = 216 respondents)

	Item	Valid n	Missing	Mean	SD	λ
*Being in control*						
1	Do you feel in control of your life?	213	3 (1%)	3.00	0.84	0.885
2	Are you able to manage your life how you like?	212	4 (2%)	2.52	0.76	0.697
3	Are you in charge of what happens in your life?	213	3 (1%)	2.80	0.81	0.611
*Avoidance of a loss frame*						
4	Are you pessimistic about your future?^a^	211	5 (2%)	3.23	0.78	0.856
5	Do you feel helpless?^a^	214	2 (1%)	3.30	0.79	0.871
6	Do you feel that your problems continue to get worse and worse?^a^	215	1 (1%)	3.15	0.87	0.846
7	Do you see opportunities to turn your life in a positive direction?	211	5 (2%)	2.42	0.85	0.801
*Efficiency*						
8	Do you find enjoyable activities easily?	212	4 (2%)	2.54	0.91	0.806
9	Do you do things that are both fun and challenging at the same time?	214	2 (1%)	2.46	0.83	0.843
10	Are your daily activities important to you for multiple reasons?	214	2 (1%)	2.93	0.80	0.774
11	Do your activities provide you with multiple benefits, such as fun, relaxation, and a social life?	212	4 (2%)	2.73	0.81	0.932
12	Are your preferred activities things you do with people who are important to you?	212	4 (2%)	2.74	0.84	0.697

^a^Reverse-coded items

### Measurement model findings

Model fit statistics revealed a large and significant chi-squared value (*χ*^2^_[51]_ = 107.928, *p* < 0.001), reflecting sensitivity to sample size [38]. The CFI (0.985) exceeded the pre-set cut-off point of 0.95, indicating small differences between the estimated and observed models. The SRMR value (0.067) was well below 0.08, suggesting good overall model fit. The RMSEA value (0.07) was slightly above the cut-off point of 0.06, indicating reasonable fit according to general consensus (Table 3) [39, 40].

**Table 3:** Model fit of the SPF-Q

*Χ*^2^ (*p*)	RMSEA	CFI	SRMR
107.928 (0.0)	0.0721	0.985	0.0670

### Internal consistency and inter-correlations

The internal consistency of the subscales was good, with Cronbach’s alpha values ranging from 0.723 (*being in control*) to 0.861 (*efficiency*; Table 4). As expected, the three subscales were moderately associated (inter-correlations ranged from 0.561 to 0.577 for the observed data and from 0.630 to 0.706 for the latent scores), indicating that the concepts were related, but distinct (discriminant validity). The associations between the latent constructs of the production needs are higher than between the observed total scale values (see Table 4) because they account for measurement error. Table 4 also reveals high associations between the observed and latent scores pointing to construct validity. Internal consistency of the 12-item SPF-Q instrument as represented by a composite reliability index based on the factor loadings of the first-order constructs, yielded a value of 0.881.

**Table 4:** Scale characteristics and (inter-)correlations for the 12-item SPF-Q

	Cronbach’s *α*	Scale mean (SD)	1	2	3
1. Being in control	0.723	2.77 (0.65)	[0.939]	0.706	0.680
2. Avoidance of a loss frame	0.839	3.03 (0.68)	0.577	[0.996]	0.630
3. Efficiency	0.861	2.68 (0.68)	0.574	0.561	[0.982]

All correlations *p* < 0.001 (two-tailed). Above the diagonal correlations for the latent scores are reported and below the diagonal correlations for the observed data. On the diagonal [between brackets] the associations between the observed and latent scores are reported

### Associations of production and substantive needs with overall well-being and life satisfaction

As expected, we observed moderately strong associations between substantive and production needs, and between production needs and overall well-being and life satisfaction (Table 5). Patients who were more in control, had avoided a loss frame, and were better able to achieve efficiency in the production of their well-being reported higher levels of overall well-being and life satisfaction. The association between production needs (the *how*) and the fulfilment of the substantive needs (the *what*) suggests that the former help people to be more productive in the realisation of well-being.

**Table 5:** Correlation of production needs with substantive needs, overall well-being, and life satisfaction

	Being in control	Avoiding a loss frame	Efficiency
*Substantive needs*			
Affection	0.378	0.298	0.391
Behavioural confirmation	0.528	0.432	0.449
Status	0.363	0.282	0.364
Comfort	0.552	0.634	0.469
Stimulation	0.679	0.608	0.727
Overall well-being	0.681	0.608	0.645
Life satisfaction	0.561	0.660	0.565

All correlations *p* < 0.001 (two-tailed)

### Production needs explain life satisfaction in addition to fulfilment of substantive needs

The expectation that production needs would explain life satisfaction in addition to the fulfilment of substantive needs was supported for *avoiding a loss frame* and *efficiency* (Table 6). Patients who perceived that they adjusted to changing circumstances and who used multi-functional activities to produce well-being efficiently had greater life satisfaction. *Being in control* did not add to the explanation of patients’ life satisfaction after substantive needs were taken into account. Only the substantive needs *comfort* and *stimulation* explained patients’ life satisfaction in the first step of the multivariate regression analysis, and the effect of *stimulation* dissipated in the second step, when production needs were added to the equation. Clearly, physical well-being and especially physical comfort are most important for these multimorbid patients.

**Table 6:** Regression of background characteristics, substantive needs, and productive needs on life satisfaction

	F-change	Beta	Beta
Substantive needs	31.541***		
Affection		− 0.045	− 0.076
Behavioural confirmation		0.083	0.016
Status		− 0.072	− 0.040
Comfort		0.532***	0.363***
Stimulation		0.217**	− 0.082
Production needs	15.001***		
Being in control			0.075
Avoiding a loss frame			0.288***
Efficiency			0.262**
Adjusted R square		0.433	0.533

^**^p < 0.01; ***p < .001 (two-tailed)

## Discussion

In this study, we demonstrated that the SPF-Q is a valid and reliable instrument for the assessment of production needs among multimorbid patients. As expected, we saw that substantive and production needs were related and, more importantly, that production needs were important in addition to substantive needs for multimorbid patients’ overall level of well-being (i.e. life satisfaction). To increase our understanding of how people achieve well-being and to determine the support and care needed to protect deterioration of their well-being, investigation of the underlying substantive as well as production needs to realise well-being is crucial. Given that multimorbidity is becoming the leading threat to population health, instruments such as the SPF-Q can help to improve the ability to achieve well-being in this vulnerable population. Confirmatory factor analyses revealed good indices of fit for the instrument. As indicated by the high reliability coefficient, the scale also showed good internal consistency. We found support for construct validity through significant positive correlations between substantive and production well-being needs, as well as with overall well-being and life satisfaction.

Regarding the bivariate associations of the production as well as substantive needs with overall well-being and life satisfaction this study found moderately strong associations between substantive and production needs, and between production needs and overall well-being and life satisfaction. These findings indicate that multimorbidity patients who were more in control, had avoided a loss frame, and were better able to achieve efficiency in the production of their well-being reported higher levels of overall well-being and life satisfaction which was in line with our expectation [10, 20]. For this population the substantive need comfort seems most important for their life satisfaction. This is in line with earlier research showing that multimorbidity is associated with poor physical well-being (e.g. worse physical functioning, poorer health outcomes and mortality) [3, 5, 6]. While in the first step of the multivariate analysis stimulation was also significantly associated with life satisfaction this effect dissipated in the second step, when the production need efficiency was added to the equation. Looking at the underlying items of efficiency and stimulation there may, however, be some overlap. Given that efficiency refers to a person’s abilities to use multi-functional activities (e.g. finding both fun and challenging activities) this also assesses a person’s engagement in stimulating activities. Not surprisingly, efficiency appeared to be more important when both needs were included in the equation.

We limited the operationalisation of production needs to *being in control*, *avoidance of a loss frame*, and *efficiency*. Other production needs that have been mentioned in the literature include *safety* (security or stability), *competence* [10], and *consistency* [20]. In our view, the need for *safety* is related closely to *loss avoidance*, or a person’s (in)capacity to adjust to changing circumstances and to protect his or her resources, which makes deterioration of the quality of production functions more likely. For example, research has shown that older people with higher levels of loss avoidance tend to have more difficulty in achieving well-being after retirement. Karpas et al. [41] clearly showed that income decline had little apparent adverse effect on the well-being of most retirees, but it did have an effect among those with high levels of loss avoidance. More secure individuals (those not caught in a loss frame) have been shown to find it easier than others to adapt to bereavement and relationship loss [7, 42, 43]. Regarding *competence* [10, 23], self-management abilities or self-regulatory skills help people to better manage their resources [44], thereby helping them to produce more well-being [45]. In contrast, the realisation of production *needs* (not merely abilities or skills) is expected to directly result in higher levels of well-being. The belief that one is capable of quitting smoking (a self-efficacy skill) is quite distinct from the ability to muster the willpower to accomplish this goal (*being in control*). Although the data suggest that *avoidance of a loss frame* and *efficiency* contribute directly to overall well-being (life satisfaction) in addition to substantive needs, the same is not true for *being in control*. Apparently, *being in control* contributes solely via the fulfilment of substantive needs, in contrast to the common belief that *control* (or autonomy) is a universal need (e.g. [23]). Further research in other study populations should be conducted to determine whether *being in control* is a skill that simply aids the production of substantive needs, or if it also contributes to well-being directly because it is a production need. We did not incorporate *consistency* in the current study, but perhaps it should be included in future as part of *efficiency*. *Consistency* or avoidance of moral conflict is likely to improve the input/output ratio (i.e. quality) of production functions [20]. Moreover, the efficient production of well-being is related not only to immediate production, but also to investment behaviour [7]. When a person’s production functions fulfil long-term as well as short-term goals, they are clearly more efficient. This aspect is in line with Lindenberg’s description of the incorporation of long-term production of well-being and improving one’s position, rather than limiting the concept to immediate gratification.

Some limitations need to be taken into consideration when interpreting our study findings. Several measurement properties of the SPF-Q could not be evaluated in this study and thus remain undefined [46]. Further research is needed to assess the instrument’s stability and reliability over time, we did not conduct test–retest reliability. Secondly, we included three production needs that are important for the well-being of multimorbid patients. Based on other research, however, other production needs may also be important; further research is needed to investigate this possibility. Thirdly, we included only multimorbid patients in our sample which may have affected our study findings. Our results showed, for example, that physical well-being and especially physical comfort seem to be most important for the life satisfaction of multimorbid patients. Assessment of quality of production functions to achieve well-being among other populations may lead to different findings. However, given the psychometric properties we do expect that the SPF-Q will be useful for the assessment of production needs in other populations as well. Further research, however, is needed to confirm this expectation.

## Conclusion

This study demonstrated that the psychometric properties of the SPF-Q are good, which makes it a promising tool for the assessment of production needs to achieve well-being. Whereas earlier research already showed the importance of fulfilment of substantive needs to achieve well-being this research shows the additional importance of fulfilment of production needs (*being in control*, *avoiding a loss frame*, and *efficiency*) to achieve well-being. Given that multimorbidity is becoming the leading threat to population health, such an instrument can help to improve the ability to achieve well-being in this vulnerable population.

## Data Availability

The dataset analyzed during the current study is available from the corresponding author on reasonable request.

## Abbreviations

COPD: Chronic obstructive pulmonary disease
SPF-Q: Social Production Function-Quality of production functions
SPF-ILs: Social Production Function Instrument for the Level of well-being short
SRMR: Standardised root mean square residual
RMSEA: Root mean square error of approximation
CFI: Comparative fit index
SD: Standard deviation
